# Poly Adenosine Diphosphate-Ribose Polymerase (PARP) Inhibitors in Pancreatic Cancer

**DOI:** 10.7759/cureus.22575

**Published:** 2022-02-24

**Authors:** Tejasvi Sunkara, Sai Samyuktha Bandaru, Rajendra Boyilla, Rajesh Kunadharaju, Prithvi Kukkadapu, Adithya Chennamadhavuni

**Affiliations:** 1 Internal Medicine, UnityPoint Health - St. Luke’s Hospital, Cedar Rapids, USA; 2 Internal Medicine, Baton Rouge General Medical Center-Bluebonnet, Baton Rouge, USA; 3 Internal Medicine, CHRISTUS St. Frances Cabrini Hospital, Alexandria, USA; 4 Department of Pulmonary and Critical Care Medicine, University at Buffalo, Buffalo, USA; 5 Internal Medicine, Huntsville Hospital, Huntsville, USA; 6 Hematology and Medical Oncology, University of Iowa, Iowa City, USA

**Keywords:** overall survival, clinical trials, brca mutation, parp inhibitors, pancreatic cancer

## Abstract

Pancreatic cancer is the third most common cause of cancer death in the United States and eleventh worldwide. The majority of patients present with advanced disease with five-year overall survival of less than 10%. Traditional chemotherapy has been the mainstay treatment for years, with limited improvement in survival. Relative success has been achieved with agents targeting the DNA damage repair (DDR) mechanisms with poly adenosine diphosphate-ribose polymerase (PARP) inhibitors. The initial benefit was observed in patients with germline *breast cancer-associated* (*BRCA*) mutations. Multiple trials are now underway exploring PARP inhibitors in other DDR mutations such as the *ataxia-telangiectasia mutated* (*ATM*) gene and the *cyclin-dependent kinase inhibitor 2A* (*CDKN2A*) gene (familial atypical multiple mole and melanoma syndrome), *mismatch repair *genes (Lynch syndrome), and others. PARP inhibitors are being evaluated as a single agent or combination chemotherapy, immunotherapy, and maintenance after chemotherapy. Here, we review current clinical trials targeting various DDR mutations and treatment strategies.

## Introduction and background

**Figure 1:**
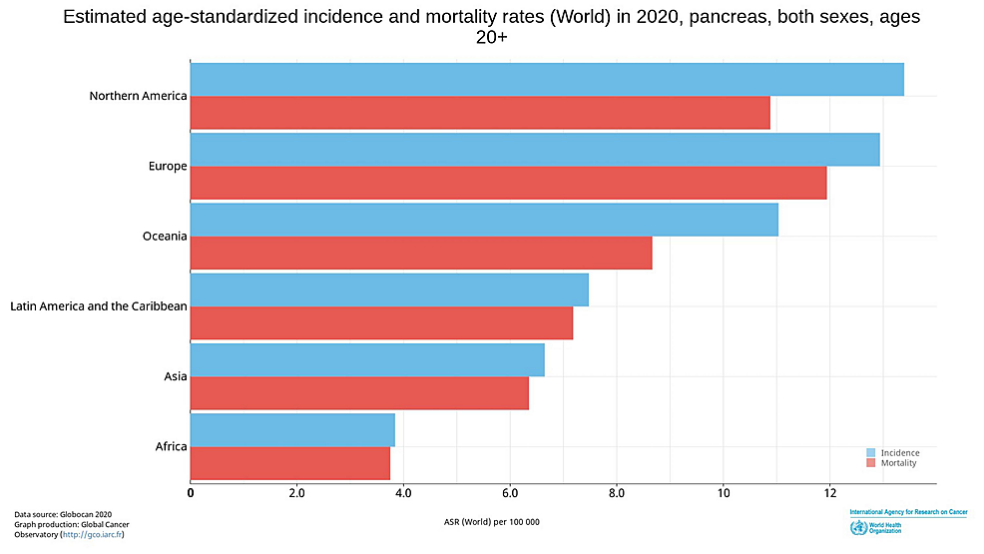
Estimated age-standardized incidence and mortality rates for the global population in 2020, aged 20+ years, including both sexes for pancreatic cancer. Sung H, Ferlay J, Siegel RL, Laversanne M, Soerjomataram I, Jemal A, Bray F: Global Cancer Statistics 2020: GLOBOCAN Estimates of Incidence and Mortality Worldwide for 36 Cancers in 185 Countries. 2021, 71:209-249. 10.3322/caac.21660 [1]. This article is available under the Creative Commons CC-BY-NC license and permits non-commercial use, distribution, and reproduction in any medium provided the original work is properly cited.

Pancreatic cancer comprises 3.2% of all new cancer cases in North America and 3% of cases worldwide. Mortality is approximately 8% of all cancer deaths in North America and 5% worldwide, making it the third most common cause of cancer death in the United States and seventh worldwide [1]. Estimated age-standardized incidence and mortality are higher among North American and European populations in comparison to the rest of the world (Figure 1) [1]. Overall, 82% of patients are diagnosed with an advanced-stage disease on presentation, with abysmal five-year overall survival of approximately 9% [2]. Traditional chemotherapy has been the mainstay treatment with limited improvement in survival, necessitating the need to identify targetable agents to significantly impact survival. Relatively successful strategies with agents targeting the DNA damage repair (DDR) mechanism, especially among patients with *breast cancer-associated* (*BRCA*) mutations, are being examined in the setting of pancreatic cancer.

Incidence of DNA damage repair genes

Pancreatic cancer is largely sporadic, with 5-20% of patients having a germline predisposition [3-7]. Genetic mutations of damaged DNA repair genes such as *BRCA1/2*, *partner and localizer of BRCA2* (*PALB2*), and the *ataxia-telangiectasia mutated* (*ATM*) gene along with the *cyclin-dependent kinase inhibitor 2A* (*CDKN2A*) gene (familial atypical multiple mole and melanoma syndrome) and *mismatch repair* genes (Lynch syndrome) are associated with significantly increased risk of pancreatic cancer. *BRCA1 *is seen in up to 2.4% of pancreatic cancer patients, with up to a three-fold higher risk of developing pancreatic cancer [3,8]. *BRCA2 *mutations are seen in about 6% in sporadic and up to 17% in familial pancreatic cancer, with an even higher incidence in the Ashkenazi Jewish population [3,7-10]. *BRCA2*-mutated patients have a 3.5-6-fold higher risk for developing pancreatic cancer [11,12]. *PALB2 *is reported to occur in 2-4.9% of familial pancreatic cancer patients and 0.5% sporadically [9,13-15]. *ATM* mutations have been reported to occur in 2.4% of familial pancreatic cancer patients [16] and 3-4% in a study with a population unselected for family history of cancer [9,17]. *Checkpoint kinase 2* (*CHEK2*) has been observed in up to 4% of patients and *CDKN2A *in 1.7% of patients [4,9,18-20]. A summary of the above mutations has been presented in Table 1.

**Table 1:** Incidence of various DNA damage repair genes. *BRCA*: *breast cancer-associated*; *ATM*: *ataxia-telangiectasia mutated*; *CHEK2*: *checkpoint kinase 2*; *CDKN2A*: *cyclin-dependent kinase inhibitor 2A* G: germline; S: sporadic

BRCA1	BRCA2	ATM	CHEK2	PALB2	CDKN2A
0.5–3.4% (G&S)	3–17% (G&S)	3–4% (G&S)	3.9% (G&S)	0.5% (S) and 2–5% (G)	1.7% (G&S)

Synthetic lethality and DNA damage repair

The synthetic lethality concept was first introduced by Bryant et al. [21] and Farmer et al. in 2005 [22], which explains synergistic genetic mutations in two or more genes leading to cellular death. There are many DNA repair mechanisms that help restore genetic integrity in cells as well as common repair pathways that sense single-stranded DNA breaks are base excision repair, nucleotide excision repair, and mismatch repair. If the single-stranded break repair is not effective, it leads to the formation of double-stranded DNA breaks. Double-stranded DNA breaks are repaired through homologous recombination (HR) during the last phase of the S and G2 phases of cell cycles and non-homologous end joining (NHEJ) during the G1 phase [23]. Based on this concept, several targeted therapies against tumor-specific gene defects have been used to kill cancer cells. Poly adenosine diphosphate-ribose polymerase (PARP) inhibitors use the concept of synthetic lethality to target *BRCA *mutations.

**Figure 2:**
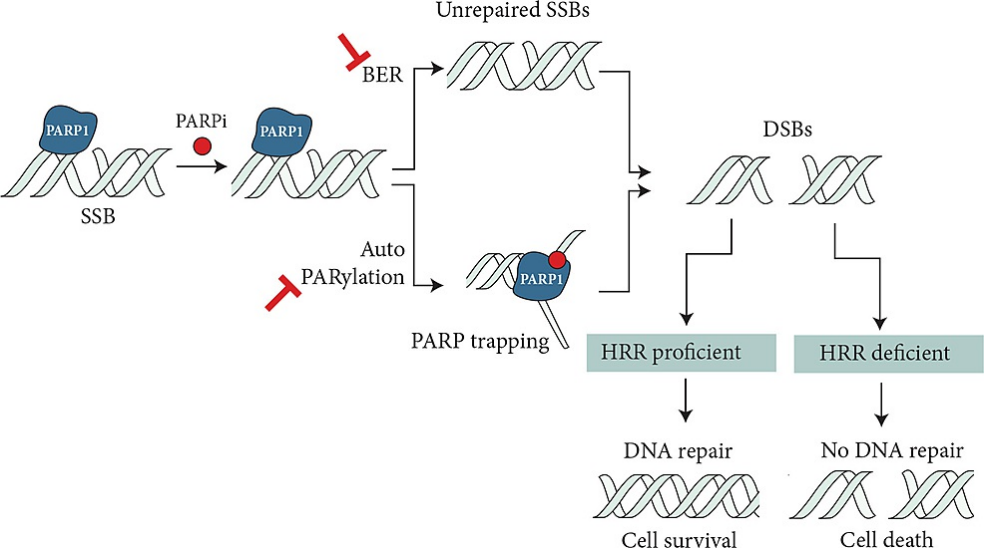
Proposed mechanism of PARP inhibitors in HRR-deficient cells. PARP inhibitors trap PARP1 through inhibition of autoPARylation with or without PARP release, which in the presence of HR deficiency leads to the accumulation of double-stranded DNA breaks and ultimately to cell death. SSBs: single strain breaks; BER: base excision repair; DSBs: DNA double-strand breaks; HRR homologous recombination repair; PARP: poly adenosine diphosphate-ribose polymerase Mateo J, Lord CJ, Serra V, et al.: A decade of clinical development of PARP inhibitors in perspective. Ann Oncol. 2019, 30:1437-47. 10.1093/annonc/mdz192 [24]. This article is available under the Creative Commons CC-BY-NC license and permits non-commercial use, distribution, and reproduction in any medium provided the original work is properly cited.

PARP1 senses DNA damage and binds to the region of DNA breaks, inducing signal transduction by producing PAR chains (autoPARylation) on target proteins. PAR chains attract DNA repair effectors, thereby competing for DNA repair [24]. PARP inhibitors trap PARP1 through inhibition of autoPARylation with or without PARP release, which in the presence of HR deficiency leads to the accumulation of double-stranded DNA breaks and ultimately to cell death (Figure 2). Cells lacking *BRCA1/2* and *PALB2 *are predominantly affected by PARP inhibition. Platinum agents create DNA cross-links, disrupting DNA functions, which is further potentiated by the lack of a DNA repair mechanism. Platinum agents are preferred in *BRCA*-mutated patients as they have been shown to significantly improve overall survival in the advanced or metastatic disease setting [25]. Single-strand breaks, which are repaired through the base excision pathway, are also inhibited by PARP inhibitors, resulting in cell death in HR-deficient cells. Multiple PARP inhibitors are currently available with different PARP trapping potencies.

## Review

PARP inhibitors

Following the detailed introduction, PARP inhibitors sensitize cancer cells to DNA damaging therapies and inhibit DNA repair mechanisms leading to synthetic lethality. Several clinical trials such as the OlampiAD study for breast cancer [26], SOLO study for ovarian cancer [27], and TOPARP-B study for prostate cancers [28] with underlying germline *BRCA1/2* mutations have shown promising results with an increase in response rate and progression-free survival (PFS). According to the National Comprehensive Cancer Network (NCCN) practice guidelines, germline testing is recommended for all patients with confirmed pancreatic cancer [29]. The effectiveness of PARP inhibitors can be examined in cancers that possess BRCAness gene defects. BRCAness can be described as tumors that do not have germline line *BRCA *mutation but have gene defects that share phenotypic similarities with *BRCA *mutations and have defective HRR. This review focuses mainly on the clinical trials of several PARP inhibitors to date and those currently underway to assess the efficacy of PARP inhibitors in the treatment of pancreatic cancer (Table 2) [30-32].

**Table 2:** Potency of various PARP inhibitors. PARP: poly adenosine diphosphate-ribose polymerase

PARP inhibitor	Potency	PARP target
Olaparib	1	PARP 1, 2, 3
Rucaparib	1	PARP 1, 2, 3
Talazoparib	100	PARP 1, 2
Veliparib	0.1	PARP 1, 2
Niraparib	2	PARP 1, 2
Pamiparib (BGB 290)	10	PARP 1, 2

Olaparib

Olaparib is currently the only Food and Drug Administration (FDA)-approved PARP inhibitor for use in pancreatic cancer. Multiple clinical trials are underway evaluating olaparib use for pancreatic cancer both as a monotherapy and combination therapy (Tables 3, 4). FDA approval was based on the POLO trial, a phase III randomized, placebo-controlled, double-blinded study that evaluated the efficacy of olaparib as maintenance therapy [33]. In this study, a total of 154 patients had germline *BRCA1 *or *BRCA2*-mutated metastatic pancreatic cancer without disease progression during at least 16 weeks of first-line platinum-based chemotherapy. In the study, 92 patients received olaparib and had significantly improved median PFS of 7.4 months versus 3.8 months in the placebo group (p = 0.004). At two years, 22.1% in the olaparib group had no disease progression in comparison to 9.6% of patients in the placebo group. However, planned interim analysis at 46% data maturation showed no difference in OS between the groups. There were certain limitations to the study. Included trial patients had a good response to the platinum agents prior to maintenance PARP treatment and non-responders were not included. Maintenance chemotherapy after six months of intense triple chemotherapy in responders is a common strategy employed with single-agent 5-fluorouracil or capecitabine versus combination with irinotecan or platinum agents, unlike placebo in the control arm. Overall, this study paved the way for multiple therapeutic and maintenance strategies in pancreatic cancer patients.

**Table 3:** Olaparib as maintenance therapy and single-agent therapy. PFS: progression-free survival; OS: overall survival; ORR: objective response rate; *BRCA: breast cancer-associated*; PDAC: pancreatic ductal adenocarcinoma; *ATM*: *ataxia-telangiectasia mutated*; *PALB2*: *partner and localizer of BRCA2*; *FANCB*: *FA complementation group B*; *PTEN*: *phosphatase and tensin homolog*; *CCNE1*: *cyclin E1*; SD: stable disease; PR: partial response; CR: complete response

Clinical trial	Study objective	N	PFS	OS	ORR	Germline mutations	Somatic mutations
Olaparib as maintenance therapy							
NCT02184195 POLO trial [33]	Phase III randomized, placebo-controlled, double-blinded study evaluating the efficacy of olaparib as maintenance therapy	154	7.4 months	No survival benefit versus placebo at 46% data maturity	20% response rate in the study group, and 10% response in the placebo group	*BRCA1 *or *BRCA2*	Excluded
Olaparib as single-agent therapy							
NCT02677038 (United States) and NCT02511223 (Israel) [34]	Parallel phase II trials in the United States and Israel evaluating the efficacy of olaparib in advanced PDAC with BRCAness with ≥ prior systemic therapy	11 patients in the United States and 21 in Israel	Median PFS of ~25 weeks in the United States and 14 weeks in Israel	Not reached	~ 80% (17/21) from the Israel group and 73% (8/11) from the US group had stable or partial response. No responses were seen in platinum-refractory cases	Excluded	DDR-genetic aberrations (*ATM*, *PALB2*, *BRCA* somatic, *FANCB*, *PTEN*, and *CCNE1*), family history of BRCA without DDR genetic aberrations
NCT01078662 [35]	Phase 2 study assessing the efficacy and safety of olaparib in confirmed genetic *BRCA1 *or *BRCA2 *mutation patients in advanced tumors with 23 pancreatic patients in the study	23	Median PFS of 4.5 months	Median OS of 9.8 months	57% (SD+PR+CR) at >8 weeks	*BRCA1 *or *BRCA2*	Excluded

**Table 4:** Olaparib as combination therapy. MTD: maximum tolerated dose; *BRCA*: *breast cancer-associated*; *ATM*: *ataxia-telangiectasia mutated*; HRD: homologous recombination deficiency; DDR: DNA damage repair

Clinical trial	Phase	Study objective	Germline mutations	Somatic mutations	Outcomes
Olaparib with chemotherapy and targeted therapy					
NCT00515866 [36]	Phase I	Evaluate the safety and MTD of olaparib with gemcitabine versus gemcitabine alone in patients with locally advanced/metastatic pancreatic cancer	Unselected for mutations		Out of 66 patients treated, olaparib 100 mg twice daily with gemcitabine 600 mg/m^2^ was the most tolerable combination.
NCT01296763 [37]	phase I	Dose escalation trial evaluating olaparib in combination with irinotecan, cisplatin, and mitomycin C in patients with advanced pancreatic cancer	Known *BRCA*-mutated or Jewish ancestry patients and those with familial and sporadic pancreatic cancer were included		Early closure due to toxicity
NCT03682289 [38]	phase II	Studying the efficacy of ATR kinase inhibitor AZD6738 alone versus combination with olaparib in patients with advanced renal cell, urothelial and pancreatic cancers	N/A	BAF250a-positive received AZD6738 with olaparib and BAF250a-negative or *ATM*-mutant received AZD6738 only	Currently recruiting
NCT02498613 [39]	Phase II	Study evaluating cediranib maleate combined with olaparib in advanced breast, non-small-cell and small-cell lung cancer, and pancreatic cancer patients	Identification of DNA repair genes in tumors using the BROCA panel with a plan to correlate tumor regression with mutations status		Currently recruiting
NCT03205176 [40]	Phase I	Study evaluating MTD and preliminary antitumor activity of AZD5153 in relapsed/refractory malignant solid tumor, including lymphoma patients as a single agent or in combination with olaparib.	Patients are included regardless of *BRCA *status. Planned to collect BRCA and HRD gene mutational status		Currently recruiting
NCT04005690 [41]	Phase II window of opportunity trial	Evaluating cobimetinib or olaparib response in patients with resectable pancreatic cancer by comparing pretreatment biopsy samples with posttreatment resection specimens	N/A		Currently recruiting
Olaparib with immunotherapy					
NCT03851614 (DAPPER) [42]	Phase II	Evaluating changes in genomic and immune biomarkers in tumor, peripheral blood, and stool; changes in radiomic profiles, with the combination of durvalumab with olaparib or cediranib	Patients with germline or somatic DDR genes will be evaluated retrospectively but is not an eligibility criteria		Currently recruiting

Olaparib as monotherapy: Two ongoing parallel phase 2 trials [34] in the United States (NCT02677038) and Israel (NCT02511223) are evaluating the efficacy of olaparib in advanced pancreatic ductal adenocarcinoma (PDAC) with BRCAness (excluding germline *BRCA1/2* mutations) after at least one prior systemic therapy regimen. Of the 11 U.S. patients, two achieved partial response (PR), six reported stable disease (SD), and three have experienced progressive disease (PD), with a median PFS up to 24.7 weeks. Of the 21 Israeli patients, five are with SD, and 12 are with PD, with a median PFS of 14 weeks. Another phase 2, open-label study assessed the efficacy and safety of olaparib in 23 advanced pancreatic cancer patients with confirmed genetic *BRCA1 *or *BRCA2 *mutations (NCT01078662) [35]. Over 74% of patients with *BRCA2 *mutations with a mean of two prior therapies with 65% receiving prior platinum. Overall, 22% of patients had a complete response (CR) or PR, and 35% had SD at eight weeks. The response was independent of *BRCA1/2* status as well as response to prior platinum treatment. Median PFS was 4.5 months with a median OS of 9.8 months at the end of the study. OS rate at 12 months was 41%.

Olaparib with chemotherapy, other targeted therapies: NCT00515866 is a phase I study of olaparib with gemcitabine in patients with advanced solid tumors to evaluate the safety and maximum tolerated dose (MTD) of the combination for pancreatic cancer treatment [36]. A total of 68 patients were included in the dose-escalation and expansion phase, with more than 81% of patients with combination treatment experiencing grade ≥3 adverse events, predominantly cytopenias (55%). Based on this study, the ideal combination was olaparib 100 mg twice daily (intermittent dosing) with gemcitabine 600 mg/m^2^ with manageable toxicities. NCT01296763 [37] is another phase 1 dose-escalation trial evaluating olaparib in combination with irinotecan, cisplatin, and mitomycin C in patients with advanced pancreatic cancer. Unfortunately, patients in this trial developed significant toxicity with this combination therapy, predominantly cytopenias, resulting in early study closure.

NCT03682289 [38] is a non-randomized phase II trial studying the efficacy of ataxia telangiectasia and Rad3-related protein (ATR) kinase inhibitor AZD6738 alone or in combination with olaparib in patients with advanced renal cell, urothelial, and pancreatic cancers. Patients tested for BAF250a positive expression on immunohistochemistry received AZD6738 in combination with olaparib. BAF250a-negative or *ATM*-mutant patients received AZD6738 only. NCT02498613 [39] is an open-label phase II trial studying cediranib maleate (vascular endothelial growth factor (VEGF)-tyrosine kinase inhibitor (TKI)) combined with olaparib in advanced breast, non-small-cell and small-cell lung cancer, and pancreatic cancer patients. Apart from the primary objective to assess the response to the treatment, other exploratory objectives include the correlation of response to DNA repair gene mutations, estimating levels of angiogenesis markers such as VEGF at baseline and after treatment, evaluating tumor hypoxia in lung cancer patients, and circulation tumor DNA measures through treatment course. This study unfortunately did not show any meaningful activity.

Other ongoing trials are NCT03205176 [40], a phase I, multicenter, dose-escalation study evaluating AZD5153 (bivalent BRD4/BET bromodomain inhibitor) pharmacokinetics, anti-tumor activity with tolerability both as a monotherapy and in combination with olaparib in patients with relapsed/refractory malignant solid tumors and lymphomas. NCT04005690 [41] is a phase II trial evaluating how cobimetinib or olaparib works in patients with resectable pancreatic cancer by comparing pretreatment biopsy samples with posttreatment resection specimens. The primary goal is to use the results in designing future biomarker-driven trials.

Olaparib with immunotherapy: NCT03851614 [42] (DAPPER) is a phase II basket study in patients with advanced pancreatic cancer, mismatch repair colorectal cancer, and leiomyosarcoma to evaluate changes in genomic and immune biomarkers in the tumor, peripheral blood, and stool samples. The study involves a combination of durvalumab (programmed death-ligand 1 (PD-L1)) with olaparib or cediranib (AZD2171, a small-molecule VEGF-TKI). This study is still ongoing, and results have not been published yet.

Veliparib

Veliparib as monotherapy: There are nine clinical studies involving veliparib (Tables 5, 6). Monotherapy was evaluated as a phase 1 study (NCT00892736) in patients with refractory *BRCA1/2*-mutated solid cancer; platinum-refractory ovarian, fallopian tube, or primary peritoneal cancer; or basal-like breast cancer. At the MTD, *BRCA*-positive patients had a response rate of 58%. This study had a limited number of pancreatic cancer patients, and specific data regarding pancreatic cancer outcomes were not available [43].

**Table 5:** Veliparib as monotherapy. *BRCA*: *breast cancer-associated*

Clinical trial	Phase	Study objective	Germline mutations	Somatic mutations	Outcomes
Veliparib monotherapy					
NCT00892736 [43]	I	Refractory *BRCA1/2*-mutated solid cancer; platinum-refractory ovarian, fallopian tube, or primary peritoneal cancer; or basal-like breast cancer	Refractory *BRCA1/2*-mutated solid cancer		*BRCA*-positive patients had about a 58% response rate. Pancreatic data are limited

**Table 6:** Veliparib in combination therapy MPC: metastatic pancreatic cancer; PFS: progression-free survival; OS: overall survival; *BRCA*: *breast cancer-associated*; *ATM*: *ataxia-telangiectasia mutated*; *PALB2*: *partner and localizer of BRCA2*; *FANCB*: *FA complementation group B*; *CHEK2*: *checkpoint kinase 2*; IMRT: intensity-modulated radiotherapy; SD: stable disease

Clinical trial	Phase	N	Study objective	Germline mutations	Somatic mutations	PFS	OS	ORR
Veliparib with chemotherapy								
NCT01908478 [44]	I	30	Evaluates veliparib (ABT-888) in combination with gemcitabine and IMRT in locally advanced and unresectable pancreatic cancer patients	None	DDR mutations, PARP level, and tumor mutation burden were once enrolled in the trial	9.8 months	mOS for DDR-deficient was 19 months and DDR-intact was and 14 months	SD was 93% (28/30), PR and 3% (1/30)
NCT01489865 [45]	I/II	64 (57 patients in the final analysis)	Evaluates veliparib in combination with FOLFOX in MPC	Trial included patients with DDR-positive and two m*BRCA2* patients		3.7 months	8.5 months	26%
						DDR positive 7.2 mo	11.1	50%
NCT01585805 [46]	II	55	Evaluates response to gem + cis in combination with beliparib (Arm – A) and without veliparib (Arm – B)	Germline *BRCA1/2* or germline *PALB2* mutation	NA	No statistically significant difference in both arms with median PFS of ~10 months and median OS of ~16 months		Insufficient power to extrapolate
NCT02890355 [47]	II	143	Evaluates response to mFOLFIRI + veliparib versus FOLFIRI alone for second-line MPC patients	15 patients: *BRCA1*, *BRCA2*, *ATM*, *FANC*, *BLM*, *SLX4*, *CHEK2*	20 patients: *BRCA2*, *PALB2*, *ATM*, *CDK12*, *FANC*, *BLM*, *POLD1*, *RIF1*, *MSH2*, *MSH6*, and other unclassified DDR.	In biomarker unselected patients, there is no difference in median PFS (~2–3 months), median OS (~5–6 months) along with increased toxicity in veliparib arm		
NCT01233505 [48]	I	17	Evaluate safety and preliminary efficacy of the combination of CAPOX with veliparib	Four breast and three ovarian are *BRCA1*-positive. Information unavailable on pancreatic cancer patient		One pancreatic cancer patient has SD		
NCT00576654 [49]	I	35	Evaluate safety and preliminary efficacy of veliparib in combination with irinotecan	Observed *BRCA *mutations carriers among ovarian cancer patient.		No pancreatic cancer patients on trial so far. PR in ~19% of patients		

Veliparib with chemotherapy: NCT01908478 is a phase I study evaluating veliparib (ABT-888) in combination with gemcitabine and intensity-modulated radiation therapy (IMRT) in patients with locally advanced and unresectable pancreatic cancer. This study among 30 patients showed a median OS of 15 months. There were no *BRCA *patients in the trial. Interestingly, median OS for DDR pathway gene-altered was 19 months and 14 months among DDR-intact patients. Increased expression of the DDR proteins PARP3 showed significantly improved OS; however, DDR pathway mutations did not correlate with OS [44]. The majority of patients had SD (93%), with PR in one patient.

NCT01489865 is a phase I/II study of veliparib in combination with 5-fluorouracil and oxaliplatin (FOLFOX) in patients with metastatic pancreatic cancer. According to the final study results, of the 64 patients, 78% were platinum-naïve, 69% had a family history, and 27% had DDR mutations. Among the total 57 evaluable patients, the objective response rate (ORR) was 26% with median PFS of 3.7 months and median OS of 8.7 months. However, in patients with DDR mutations, ORR (58%) and m PFS (7.2 months) doubled with improvement in OS (11.1 months). The responses further improved in patients with family history and platinum-naïve disease along with DDR deficiency, which appears to be a promising combination with respect to the safety and response in metastatic PDAC [45]. An earlier report from the trial mentioned, two *BRCA2 *patients were included in the study, one had a PR at 17 months, and the other had CR with normalization of carbohydrate antigen 19-9 at 10 months.

NCT01585805 is a randomized, phase II study of gemcitabine, cisplatin without (Arm A) or with (Arm B) veliparib, and a phase II single-arm study of single-agent veliparib in patients with *BRCA*- or *PALB2*-mutated pancreatic adenocarcinoma. Among the enrolled 55 patients, median PFS (~10 months) and median OS (~16 months) were not significantly improved with the addition of veliparib [46].

NCT02890355 is a randomized, phase II study evaluating second-line modified FOLFIRI with veliparib versus FOLFIRI. Out of 143 patients included in the analysis, 30% had DNA repair gene abnormalities and 9% has HR deficiency. Interim futility analysis at 35% of expected PFS events showed that the veliparib arm was unlikely to be superior to control in the biomarker unselected population. Median PFS was approximately two to three and median OS was five to six months. Biomarker-driven efficacy is yet to be published [47]. NCT01233505 is a phase I study evaluating veliparib in combination with oxaliplatin and capecitabine in advanced solid tumors. The trial included one patient with pancreatic cancer who had SD with this combination [48].

NCT00576654 is a phase I study designed to evaluate the safety and preliminary efficacy of veliparib (ABT-888) in combination with Irinotecan among advanced solid tumors. Safety of combination was established, and PR is observed in approximately 19% of patients. However, no pancreatic cancer patients were reported in the study [49].

Rucaparib

According to the literature, four clinical trials have been reported, two as monotherapy, and two as combination therapy. NCT03140670 is a phase II trial of rucaparib evaluating patients with advanced pancreatic cancer with known germline or somatic *BRCA *or *PALB2 *mutations who had not progressed on at least 16 weeks of platinum treatment. Out of 24 patients enrolled, 13 had germline *BRCA2*, three had germline *BRCA1*, two had germline *PALB2*, and one had somatic *BRCA2* mutation. Median PFS was 9.1 months from starting rucaparib therapy, with an ORR of 36.8% and disease control rate (CR + PR + SD) of 89.5% for at least eight weeks [50]. Overall, the study showed encouraging results that maintenance strategy not only for germline *BRCA*,as in POLO trial, but in other germline and somatic mutations might be an effective strategy.

NCT02042378 (RUCAPANC) evaluated the efficacy of rucaparib monotherapy in patients with pancreatic cancer with deleterious *BRCA *mutations. Sixteen of nineteen *BRCA1/2* mutations were germline, and three were somatic *BRCA2*. There were two PR, one CR, with an ORR of 15.8%, and further enrollment was halted in view of the poor response rate [51].

Rucaparib with chemotherapy: NCT03337087 [52] is a phase Ib/II trial evaluating safety along with the efficacy of liposomal irinotecan and fluorouracil with rucaparib in patients with advanced pancreatic, colorectal, biliary, and gastroesophageal cancers. Patients with pancreatic cancer could have received up to two lines of prior therapy. This is planned to evaluate responses based on the HRD mutation status. NCT04171700 [53] is a phase II study of rucaparib as a treatment for solid tumors with deleterious HRD mutations including pancreatic cancer patients. Currently, no published data are available for both studies.

Rucaparib with other targeted therapies: NCT02711137 [54] is an open-label phase I/II dose-escalation/expansion along with safety and tolerability study of INCB057643 bromodomain and extraterminal (BET) inhibitor as a single agent and in combination with multiple interventions, including rucaparib in patients with advanced malignancies. The study is currently terminated in view of safety issues. Table 7 lists the trials involving rucaparib as maintenance therapy, monotherapy, and combination therapy.

**Table 7:** Rucaparib as maintenance therapy, monotherapy, and combination therapy. PFS: progression-free survival; OS: overall survival; ORR: objective response rate; *BRCA*: *breast cancer-associated*; *PALB2*: *partner and localizer of BRCA2*; HRD: homologous recombination deficiency

Clinical trial	Phase	N	Study objective	Germline mutations	Somatic mutations	PFS	OS	ORR
Rucaparib as maintenance therapy								
NCT03140670 [50]	II	24	Evaluate maintenance in pancreatic adenocarcinoma not progressing for at least 16 weeks of platinum treatment	13 germline BRCA2, 3 germline BRCA1, 2 germline PALB2	1 somatic BRCA2	9.1	N/A	37%
Rucaparib as monotherapy								
NCT02042378 [51] RUCAPANC	II	19	Evaluation monotherapy in advanced pancreatic cancer patients with germline or somatic BRCA patients who received upto two lines of prior chemotherapy.	12 germline BRCA2, 4 germline BRCA1	3 somatic BRCA2	Halted enrollment (ORR of 16% ) in view of insufficient response rate among the first 15 patients		
Rucaparib with chemotherapy								
NCT03337087 [52]	Ib/II	N/A	Evaluate safety and preliminary efficacy of liposomal irinotecan and fluorouracil with rucaparib in patients with metastatic gastrointestinal cancers including pancreatic cancer	Plan to evaluate response based on HRD mutations	Currently enrolling			
NCT04171700 [53] LODESTAR	II	N/A	Evaluating rucaparib therapy in HRD deficient solid tumors including pancreatic cancer	HRD-deficient tumors	Currently enrolling			
Rucaparib in combination with targeted therapy								
NCT02711137 [54]	I/II	N/A	Safety and BET inhibitor as single agent and in combination with multiple interventions including rucaparib in advanced malignancy pts	Study is currently terminated in view of safety issues.				

Talazoparib

Talazoparib is a selective oral PARPi that is more potent than earlier PARP inhibitors (Table 8). NCT01286987 is a phase I, first-in-human study of talazoparib in patients with advanced or recurrent solid tumors to evaluate antitumor activity and MTD of talazoparib. The study included 13 patients with pancreatic cancer; four out of 13 patients have shown clinical benefit (CR + PR + SD = 31% ≥16 weeks), with 20% (two patients) having PR. Among patients with PR, one had *BRCA2 *and another had *PALB2 *mutations. The maximum tolerated dose was found to be 1 mg/day [55]. NCT03637491 [56] is an ongoing phase 1b/II study evaluating the safety and efficacy of avelumab, binimetinib, and talazoparib combination in patients with locally advanced or metastatic *Ras*-mutant solid tumors including pancreatic cancer patients. DDR mutations will be assessed at baseline. Objective response and dose-limiting toxicities are considered as primary outcomes. Unfortunately, the study was terminated as there was limited antitumor activity, and reaching target study drug dose levels was not feasible.

**Table 8:** Talazoparib as monotherapy and combination therapy. *BRCA*: *breast cancer-associated*; *PALB2*: *partner and localizer of BRCA2*; DDR: DNA damage repair; PFS: progression-free survival; OS: overall survival; ORR: objective response rate

Clinical trial	Phase	N	Study objective	Germline mutations	Somatic mutations	PFS	OS	ORR	
Talazoparib monotherapy									
NCT01286987 [55]	I	13	Evaluate safety and preliminary efficacy in advanced or recurrent solid tumors and study has 13 pancreatic cancer patients	*BRCA2* *PALB2*	-	N/A		20%. Patients with response were bearing DDR mutations	
Talalzoparib with combination with immunotherapy and targeted therapy									
NCT03637491 [56]	Ib/II		Evaluate and efficacy of avelumab, binimetinib and talazoparib combinations in patients with locally advanced or metastatic Ras-mutant solid tumors including pancreatic cancer pts	DDR mutations will be assessed at baseline		Currently terminated			

Niraparib

There are three ongoing trials of niraparib (two monotherapy and one combination therapy trial). NCT03601923 [57] is a phase 2, proof-of-concept trial evaluating the safety and efficacy of niraparib in patients with advanced pancreatic cancer with germline and somatic HRD mutations. Included patients received at least one line of treatment for their cancer and did not have cancer progression on an oxaliplatin regimen (similar to FOLFIRINOX or FOLFOX). Palliative radiation will be started at least a week before the initiation of niraparib. PFS is the primary outcome of the study, and overall response and survival rate are the secondary outcomes. NIRA-PANC (NCT03553004) [58] is a phase II trial evaluating the efficacy of niraparib in metastatic pancreatic cancer patients with germline or somatic HRD mutations who received at least one prior line of therapy. Currently, patients are actively being enrolled. ORR (overall response to therapy at eight weeks) is the primary outcome, and PFS and OS are the secondary outcomes. Currently, no published data are available for these studies.

Niraparib as combination therapy (with immunotherapy): Parpvax (NCT03404960) is a phase Ib/II study of niraparib plus ipilimumab (phase 2), niraparib plus nivolumab (phase 1) evaluating the safety, effectiveness, and antitumor activity (preventing tumor growth) in patients with advanced pancreatic cancer whose disease has not progressed on platinum-based therapy for at least 16 weeks [59]. PFS is the primary outcome of this trial. This study is actively recruiting patients (Table 9).

**Table 9:** Niraparib as monotherapy and in combination therapy. PFS: progression-free survival; OS: overall survival; ORR: objective response rate; *BRCA*:* breast cancer-associated*; *ATM*: *ataxia-telangiectasia mutated*; *PALB2*: *partner and localizer of BRCA2*; HRD: homologous recombination deficiency

Clinical trial	Phase	N	Study objective	Germline mutations	Somatic mutations	PFS	OS	ORR
Niraparib monotherapy								
NCT03601923 [57]	II		Proof-of-concept trial evaluating niraparib in patients with HRD pancreatic cancer progressed on one line of therapy except platinum agents	Germline and somatic mutations in BRCA1, BRCA2, PALB2, CHEK2, or ATM		Currently enrolling		
NCT03553004 NIRA-PANC [58]	II		Efficacy in metastatic pancreatic cancer patients with HRD mutations who received at least one prior line of therapy.	Germline and somatic HRD mutations		Currently enrolling		
Niraparib with immunotherapy								
NCT03404960 Parpvax [59]	Ib/II		Study of niraparib plus either ipilimumab or nivolumab in patients with advanced pancreatic cancer whose disease has not progressed on platinum-based therapy	HRD will be identified after enrollment		Currently enrolling.		

Discussion

Pancreatic cancer continues to be one of the most difficult cancer to treat with chemotherapy as the predominant first-line treatment. Immunotherapy was granted accelerated FDA approval in 2017 for MSI-high/MMR-deficient advanced solid tumor patients, and larotrectinib/entrectinib is currently approved for neurotrophic tyrosine receptor kinase gene fusion-positive patients. These therapies are currently recommended by the NCCN guidelines in patients with poor performance as firstline [29]. Targeting the DNA repair mechanism is one of the novel approaches in the treatment of pancreatic cancer, and PARP inhibitors are at the forefront of that approach. Apart from germline *BRCA1/2* patients, multiple genetic defects affecting the DNR repair mechanism mentioned as BRCAness are currently under investigation in several cancers with PARP inhibitor therapy.

The most significant success, along with the first FDA approval, was through olaparib (POLO trial) as maintenance therapy in platinum-sensitive germline *BRCA*-mutated patients. Maintenance rucaparib (NCT03140670) [50] and niraparib (NCT03553004) [58] are currently being evaluated through phase II clinical trials in patients with not only germline mutations but expanding it to somatic HRD mutations. Early results are optimistic, and these trials have the potential to further expand the patient population who can receive significant benefits through maintenance therapy.

PARP inhibitors as monotherapy were assessed among germline *BRCA1/2*-mutated ovarian cancer patients with olaparib (NCT01078662) [35], which showed durable response rates regardless of platinum sensitivity. Interestingly, two parallel phase II studies (NCT02511223 and NCT02677038) [34] studied olaparib as monotherapy in germline *BRCA1/2*-negative patients with DDR deficiency and showed a response in platinum-sensitive patients. Talazoparib in a phase Ib trial showed a good response in 20% of patients among germline *BRCA1/2*-mutated patients [55]. Rucaparib [51] in germline and somatic *BRCA *patients and veliparib [43] in germline *BRCA *or *PALB2* mutations did not show significant response when used a monotherapy. Niraparib is currently under evaluation in two trials with germline and somatic HRD mutation patients with results yet to be published [57,58].

PARP inhibitors and multiple chemotherapy combinations have been evaluated with mixed results so far, and the majority of trials are still under evaluation. Veliparib, in combination with FOLFOX among metastatic pancreatic cancer patients, shows good tolerability with combination therapy, especially among patients with platinum-naïve, DDR deficiency, and patients with a family history. However, the efficacy of veliparib needs to be evaluated in a randomized fashion [44]. Veliparib with gemcitabine and cisplatin in *BRCA*/*PALB2*-mutated patients as well as in combination with FOLFIRI as second-line therapy among DDR deficiency patients did not show any significant benefit from the addition of veliparib [45,46]. There is also increased toxicity with combination therapy, as seen in NCT01296763 [37], of olaparib with irinotecan, cisplatin, and mitomycin.

Combination with immunotherapy, along with additional targeted therapy, is one of the new strategies under development. PARPVAX [59] trial is currently evaluating the combination of niraparib with nivolumab in patients with advanced pancreatic cancer who have no disease progression with at least 16 weeks of platinum. Other combination trials such as the DAPPER [60] trial evaluating durvalumab (anti-PD-L1) with olaparib and NCT03637491 [56] trial evaluating avelumab (anti-PD-L1) with talazoparib are currently under investigation. Combination with targeted therapy is currently under evaluation with multiple agents. Trials in combination with olaparib, such as NCT03682289 [38] with AZD6738 (ATR kinase inhibitor), NCT03205176 [40] with AZD5153 (bivalent BRD4/BET bromodomain inhibitor), and NCT02498613 [39] with cediranib maleate (VEGF-TKI) are under investigation. The safety of such combinations could be a predominant issue, as seen in NCT02711137 [54], evaluating BET inhibitor as a single agent and in combination with multiple interventions, including rucaparib, currently terminated in view of safety issues.

Multiple resistance mechanisms have been hypothesized, leading to the failure of PARP inhibitors such as secondary mutations in *BRCA *genes resulting in the restoration of DNA repair mechanism, efflux pumps resulting in reduced intracellular PARP concentrations, epigenetic modifications, and loss of PARP expression, to mention a few [61]. Multiple newer PARP inhibitors are currently under development to overcome such resistance mechanisms. Other strategies, such as combination and sequential therapies, are more likely to improve the efficacy of PARP inhibitors.

## Conclusions

Pancreatic cancer has an abysmal prognosis and chemotherapy has been the mainstay of treatment with limited improvement in survival. PARP inhibitors, which work by targeting the DDR mechanism, especially patients with *BRCA* mutations, are currently approved for maintenance therapy in pancreatic cancer. Beyond maintenance therapy, it is very encouraging to see PARP inhibitors emerging as a new therapeutic option as monotherapy in combination with chemotherapy, immunotherapy, and targeted therapies. Multiple trials are also evaluating the response to therapy beyond germline *BRCA *mutation patients. Such clinical trials on the application of PARP inhibitors and their efficacy with combination therapy will be critical to improving patient outcomes.
